# Carcass Characteristics, Digestive System Traits of Spent Broiler Breeder and Dual-Purpose Hens

**DOI:** 10.3390/ani12101320

**Published:** 2022-05-22

**Authors:** Karol Włodarczyk, Dariusz Kokoszyński, Mohamed Saleh, Dariusz Piwczyński

**Affiliations:** 1 Department of Animal Breeding and Nutrition, Bydgoszcz University of Science and Technology, 85084 Bydgoszcz, Poland; karwlo19@gmail.com; 2 Department of Poultry Production, Sohag University, Sohag 82524, Egypt; bydg2016@gmail.com; 3 Department of Animal Biotechnology and Genetics, Bydgoszcz University of Science and Technology, 85084 Bydgoszcz, Poland; darekp@pbs.edu.pl

**Keywords:** dual-purpose hens, broiler breeder, carcass characteristics, digestive system, internal organ

## Abstract

**Simple Summary:**

The success achieved in the breeding of laying hens and broilers resulted in modern breeding systems. Obtaining meat from laying hens is not a good alternative to intensive broiler production. However, it can be profitable to use this type of production on organic or backyard farms. Dual-purpose hens can be an economical alternative in some production conditions. The aim of the study was to determine the differences between the features of carcasses and the digestive systems of dual-purpose hens and broiler breeders. The compared dual-purpose hens differed significantly (*p* < 0.05) from broiler breeders in terms of carcass weight and dimensions. Multipurpose hens had significantly lower (*p* < 0.05) percentages of breast muscles, leg muscles, skin with subcutaneous fat, wings, and carcass remainders. On the other hand, broiler breeders were characterized by significantly greater (*p* > 0.05) total intestinal length, duodenum, jejunum, ileum, and terminal intestine. Significant differences were noted in terms of the diameter of individual intestinal segments, with the exception of the iliac and cecum intestines. Broiler breeder hens also had a greater mass of internal organs compared to the dual-purpose layer hens.

**Abstract:**

Raising dual-purpose hens for meat is believed to bring more economic benefits to farmers selling products directly to consumers. The aim of the study was to determine the differences between the carcass features and the digestive system of multipurpose hens and spent broiler breeders. In the experiment, 20 carcasses of 70-week-old Rosa 1 dual-purpose hens and 20 carcasses of 62-week-old Ross 308 broiler breeders were used. Measurements of the length of various sections of the intestine and the diameter of individual intestinal segments were made. During the gutting, proventriculus, gizzard, liver, heart, and spleen were separated and then weighed. Dual-purpose hens differed significantly (*p* < 0.05) from broiler breeders in weight and carcass dimensions. Multipurpose hens were characterized by significantly lower (*p* < 0.05) percentages of breast muscles, leg muscles, skin with subcutaneous fat, wings, and carcass remainders. Broiler breeders were characterized by significantly longer (*p* > 0.05) total intestinal length, duodenum, jejunum, ileum, and terminal intestine. Significant differences were confirmed in terms of the diameter of some segments of the intestine. Broiler breeder hens also had a greater mass of internal organs compared to the dual-purpose layer hens.

## 1. Introduction

The meat industry in Poland is based, among others, on poultry, in particular young slaughter birds (broiler chickens and turkeys) [1]. Eggs and poultry meat are popular foods accepted by consumers all over the world [2,3]. The rapidly growing poultry meat industry causes a loss of biodiversity and limits the maintenance of local populations [4,5]. The production of male chicks of laying hens is unprofitable due to the low growth rate and low slaughter efficiency. The killing of males of this line raises more and more controversy in society. The breeding success achieved in the case of laying hens and broilers resulted in modern breeding systems. Dual-purpose hens can be an economical alternative in some production conditions [6,7,8]. However, production of this type of hen is difficult because until sex is recognized they are fattened like broiler chickens for meat production [9]. Additionally, a negative correlation was found between egg production (small size) and body composition (low content of breast muscles) in multipurpose hens, compared to hens intended for egg or meat production. [10]. Therefore, a reduced efficiency in both laying and fattening is to be expected for dual-purpose hens [11,12], resulting in a lower profit for farmers and a higher price for consumers compared to conventional egg and meat genotypes. The studies by Puchała et al. [13] showed that dual-purpose poultry production is of marginal importance. Obtaining meat from laying hens is not a good alternative compared to the intensive production of broilers. However, it can be profitable to use this type of production on organic or backyard farms. Producing dual-purpose chickens is expected to bring more economic benefits to farmers selling the products directly to consumers who are willing to pay a higher price for regional meat and eggs [7]. However, the benefits of such strategies compared to slow-growing open-range broilers in light of consumer expectations have not been assessed [14]. Moreover, Busse et al. [15,16] showed that over 80% of German consumers do not know about the production of general-purpose hens. In countries where the production of white-shelled hen eggs predominates (including the USA, Germany, Brazil, and Mexico), dual-purpose hybrids of hens are relatively little known. On the other hand, hybrids of these hens (multipurpose hens) are commonly used in the production of brown-shelled eggs, which is dominant in many countries of the world, e.g., in Great Britain, France, Poland, and Hungary. Brümmer et al. [17] showed that in recent years, consumers pay more attention to the conditions of breeding with price and taste factors being placed consecutively. Modern consumers expect meat to be tender and juicy, with good taste and lower cholesterol level. The characteristics of meat and its quality depend on many factors, including nutrition, genotype, and rearing system [13,18,19]. The studies by Hafid et al. [20] showed that age at the time of slaughter has a significant influence on the tenderness of the meat.

The digestive system includes the digestive tract and the glands that develop from its epithelial wall: salivary, mucous, intestinal, liver, and pancreas. The structure of the digestive system of poultry indicates a fast pace of digestive processes. The length of the intestines in relation to the body length in chickens and turkeys is 5–6:1, and in ducks and geese is 4–5:1 (while in pigs is 25:1, horses 20:1) [21,22,23]. The structure of chickens’ digestive system has been the subject of many studies among scientists. It is proven that in the days following hatching, weight of the small intestine increases more rapidly in relation to body weight than other organs or tissues [24]. Uni et al. [25] observed that within 4 days of hatching, the intestinal mass increased fourfold, and the maximum value of the mass of internal organs was achieved between the 3rd and 8th day of life. The structure of the poultry digestive tract undergoes processes of adaptation to changes resulting from intensive breeding and the rapid growth rate of slaughter birds or high-production laying hens and breeding birds [26,27,28].

Both the small number of scientific studies on the comparisons of dual-purpose hens and broiler breeders and the different production uses of the birds were an incentive for this study. The aim of the study was to determine the differences between the carcass features and the digestive system of female multipurpose hens and female spent broiler breeders.

## 2. Materials and Methods

The experiment used 20 Rosa 1 dual-purpose hen layer carcasses (Rhode Island Red and Sussex crossbreeds) aged 70 weeks and 20 Ross 308 broiler breeder hen carcasses aged 62 weeks.

Both groups of hens were kept on a straw-covered ground in a closed building without windows at 19–20 °C. The birds were kept in enclosed housing in accordance with the requirements given in Nutrition and Feed Management for Ross 308 Parent Stock [29]. The environmental parameters were regulated. In both cases, daylight lasted for 14 h (40–60 lux). The rearing was carried out in an intensive system. The hens were fed complete diets. Feeding ROSA 1 included 11.3–11.6 MJ ME/kg feed, 15–17% crude protein, calcium level 3.6 to 4.0%, available phosphorus 0.33 to 0.37%. Feeding Ross 308 included 12 MJ ME/kg feed, 13% crude protein, calcium level 3.2 to 3.6%, available phosphorus 0.36%. Slaughter date was related to the date of herd liquidation. Due to the lack of roosters in herd Rosa 1, only female carcasses were used for the research. The research was approved by the Ethical Committee (No. 21/2014, of 26 June 2014).

### 2.1. Carcass Weight and Measurements

Eviscerated carcasses were chilled in a refrigerated cabinet (Hendi, Gądki, Poland) at 4 °C for 18 h, and then weighed individually with an electronic scale (WLC 6/12/F1/R, Radwag, Radom, Poland) with an accuracy of 0.1 g. Using a dressmaker’s tape with an accuracy of 1 mm body length (between the first cervical vertebra and the posterior edge of the ischium), trunk length (between the tuberosity of the shoulder joint and the posterior edge of the ischium), chest circumference (behind the wings, through the anterior edge of the keel and the middle segment of the thoracic vertebrae), keel length (from the anterior to the posterior edge of the sternum), drumstick length (between the knee joint and the tarsal joint), and shank length (between the tarsal joint and the posterior surface of the outer toe at its base) were measured.

The carcasses were dissected according to the method developed by Ziołecki and Doruchowski [30]. Each carcass was dissected into breast muscles (Pectoralis major plus Pectoralis minor on both sides of the breast part), leg muscles (all muscles from both thigh and drumstick), skin with subcutaneous fat (without wing skin), abdominal fat, neck, wings, and the remainder of the carcass. The dissected components were weighed with the same electronic scale (WLC 6/12/F1/R, Radwag, Radom, Poland.

### 2.2. Digestive System Characteristics

A dressmaker’s tape was used to measure different intestine segments (duodenum, jejunum, leum, collectively, caecum, and colon) to the nearest 0.1 cm. The length of the duodenum was measured from the pylorus to the pancreatic loop, the length of jejunum from the pancreatic loop to the Meckel’s diverticulum, and the length of ileum from the Meckel’s diverticulum to the ileocaecal junction. The length of the caeca was measured from the mouth of the ileum to the vertex of the right and left caecum. The length of the colon was measured as the distance from the mouth of the caeca to the cloaca. The diameters of individual segments—the anterior, middle, and posterior parts of duodenum, jejunum, and ileum, collectively, caecum, and colon—were measured with electronic calipers to the nearest 0.01 mm. During evisceration, the gizzard, proventriculus, heart, spleen, and liver were separated and weighed to within 0.01 g on an electronic scale (PS 1000/R2, Radwag, Radom, Poland).

### 2.3. Statistical Analysis

Statistical analysis was performed of data on body weight and dimensions, percentage of individual carcass components, length and diameter of intestinal segments, and the weight of other selected internal organs. The arithmetic means and standard deviation (sd), standard error of mean (SEM), and *p* values for each feature were calculated. The analysed traits were statistically characterized using SAS software ver. 9.4. [31]. Significant differences (at *p* < 0.05) between multipurpose hens and broiler breeder hens were determined with the t-test. For all the analysed traits of the carcass, the individual bird was the experimental unit.

## 3. Results

### 3.1. Carcass Weight and Measurements

In the experiment, the differences between weight and dimensions of the carcasses of dual-purpose female hens and female broiler breeders were checked. Female dual-purpose hens differed statistically significantly (*p* < 0.05) from female broiler breeders in carcass weight (less weight) and carcass length (shorter than meat hens). Trunk length, chest circumference, keel length, drumstick length, and shank length also had lower values in dual-purpose hens than in broiler breeders (Table 1).

### 3.2. Carcasses Composition

Determining the percentage of carcass elements obtained during dissection was the next stage of this research. Dual-purpose hens were characterized by statistically significantly lower (*p* < 0.05) percentages of breast muscles, leg muscles, skin with subcutaneous fat, wings, and carcass remains. No statistically significant differences (*p* > 0.05) were found in the percentage of the abdominal fat and neck in gutted carcasses with neck (without giblets) (Table 2).

### 3.3. Digestive System

The next stage of this experiment was to compare female Rosa 1 multipurpose hens with female Ross 308 broiler breeders in terms of length and diameter of particular sections of the intestine (Table 3). Female broiler breeders were characterized by significantly longer (*p* > 0.05) total intestinal length, duodenum, jejunum, iliac intestine, and colon length. No statistically significant differences (*p* > 0.05) were found in the length of cecum, which was longer in broiler breeders. Significant differentiation was also found for the diameter of some sections of the intestine. In broiler breeders, a significantly larger (*p* < 0.05) diameter of the duodenum, jejunum, and colon was found. There were no statistically significant differences (*p* > 0.05) in the diameter of the ileum and cecum.

### 3.4. Mass of Internal Organs

Statistically significant differences (*p* < 0.05) were found in the mass of other examined internal organs. Broiler breeders had a heavier proventriculus, gizzard, liver, heart, and spleen (Table 4). Pathological changes were observed among the livers of meat hens (almost cream-colored colour, with too much liver fat, soft structure, overgrown) which resulted in an especially significant difference in the weight of this organ between general-use and meat hens.

## 4. Discussion

In our study, authors investigated only one flock per hybrid. In the studies by Siekmann et al. [10], the carcass weight of 64-day-old Lohmann Dual multipurpose roosters was 1415.7 g, while in our studies the weight of 70-week-old Rosa 1 multipurpose hens was 1234.8 g. In contrast, in the studies of Lambertz et al. [7] the weight of 75-week-old female Bresse-Gualoise carcasses (the production of this breed is intended for meat) was 1.878 g, while the carcasses of female Bresse-Gualoise x New Hampshire hens (a cross for the production of multipurpose hens) at 75 weeks of age weighed 1.836 g. In the case of studies by Siekmann et al. [10], the greater weight of animal carcasses as compared to dual-purpose birds, younger than birds in our own studies, is due to the differences in the birds’ sex. Włodarczyk et al. [32] studied 36-week-old grey partridges. The shank length of males was 5.4 cm while for females it was 5.2 cm. In the case of our research, these results differ due to the different species of poultry. In female dual-purpose hens, this length was 10.6 cm, while in female broiler breeders it was 11.4 cm. Puchała et al. [33] obtained similar results for carcass weight of 56-week-old Rhode Island hens. It amounted to 1472 g. In the case of our research, the weight was 1234.8 g, while in the broiler breeders it was 3123.0 g.

Lambertz et al. [7] obtained 31.5% breast part (without skin) in carcasses of 75-week-old Bresse-Gualoise broiler breeder females and 32.3% of the breast in carcasses of 75-week-old dual-purpose females (Bresse-Gualoise x New Hampshire). The same authors obtained 18.4% of leg parts in carcasses of broiler breeder females and 16.9% of leg parts in female dual-purpose breeds. The results also differ with regard to the percentage of wing mass: in broiler breeder females it was 10.0%, while in dual-purpose females it was 10.2%. The results differ from those obtained from our study, which may be caused by a different age of the animals during slaughter and the differences in the component composition of the feed. In the study by Puchała et al. [33], the percentage of pectoral muscles in the carcasses of 56-week-old Rhode Island multipurpose hens was 14.97%, and the percentage of leg muscles was 19.79%. In our study, these values were 15.3% and 18.4% in dual-purpose hens (comparable results) and 23.7% and 22.5% in broiler breeders, respectively (such different results are related to the different direction of use of the hens). In the same study, the proportion of abdominal fat was also determined. It constituted 6.61% of the carcass. In our research, the abdominal fat constituted 2.1% of the gutted carcass weight in female dual-purpose hens and 2.7% in female broiler breeder. In the study by Vergas-Ramell et al. [34], the percentage of pectoral muscles in 72-week-old laying hens was 19.71% (in Mos hens) and 15.43% (in Isa Brown hens). The percentage of wings in the Mos breed was 9.63% (a result similar to our own research in broiler breeders) while in the Isa Brown breed was 11.32% (a result similar to our research, in dual-purpose hens). In the studies of Rizzi et al. [35], 44-week-old Ermellinata di Rovigo laying hens showed a 16.59% share of the pectoral muscles and 10.22% of the wings in relation to the carcass weight. The results are similar to those from our own research.

Alshamy et al. [36] showed that the length of the total intestine in Ross 308 broiler chickens increased by 4.57 cm/day in the first days of life. In contrast, in dual-purpose Lohmann Dual it grew by 2.98 cm/day. The greatest increase in the length of the intestine is observed in the first week after hatching. The length of the duodenum on the first day of life of the Steggles x Ross (F1) chickens was 0.9 cm, while on day 21 it was 1.3 cm. The same relation was noticed in the case of jejunum (length 1.3 cm on the first day of life and 1.7 cm on day 21). Noticeable differences resulting from a different bird species can also be noticed in the studies by Kokoszyński et al. [37]. The total length of the intestines of King pigeons raised for meat after three breeding seasons was 120.5 cm, while in the birds raised for racing it was 105.4 cm. In our research, only females were included in the study, and their total intestinal length was 212.9 cm in dual-purpose hens and 254 cm in broiler breeders.

In the research by Kokoszyński et al. [38], in which King pigeons were studied after three reproductive seasons, it was found that the weight of the male heart was 7.3 g. In our study, only a slightly higher value of a dual-purpose female’s heart (7.6 g) was obtained. The heart weight of female broiler breeder was 16.1 g, which proves the different breeding purpose of these animals. Iji et al. [38] showed in an interesting process of how the mass of internal organs changes depending on the age of the birds. On day 1 of life of Steggles × Ross (F1) chickens, the weight of the gizzard was 11 g/100 g of body weight and on day 21 the weight was 3.9 g/100 g of body weight (in our own study, the gizzard of 70-week-old dual-purpose hens was 33.1 g, while 62-week-old broiler breeders were 38.9 g). Akinwumi et al. [39] compared four poultry layer types after a year of production. The liver spent commercial layer weight was 18.8 g, gizzard weight 30.22 g, proventriculus weight 4.72 g, spleen weight 0.94 g, and heart weight 3.77 g. The values obtained in our own research differ in the case of the liver (higher, both in female dual-purpose and female broiler breeders), proventriculus (higher values were obtained in our research), spleen (higher values were obtained in our research), and heart (in our research higher values obtained). Similar results were obtained with gizzards.

## 5. Conclusions

There are significant (*p* < 0.05) differences between female dual-purpose hens and female broiler breeders after the reproductive season in terms of carcass weight and dimensions and the percentage of carcass elements, as well as the length and diameter of individual intestinal segments and the weight of internal organs. These differences mainly result from the different directions of selection because of different uses of the compared hybrids of commercial hens.

## Figures and Tables

**Table 1:** Carcass weight and dimensions in dual-purpose hens and broiler breeder hens after first egg production season.

Trait	Genotype		SEM	*p*-Value
	Dual-Purpose Hens (*n* = 20)	Broiler Breeder Hens (*n* = 20)		
Carcass weight (g)	1234.8 ± 164.3 ^b^	3123.0 ± 244.8 ^a^	154.8	<0.001
Carcass length (cm)	30.0 ± 1.2 ^b^	33.6 ± 2.8 ^a^	0.4	<0.001
Trunk length (cm)	19.1 ± 0.4 ^b^	21.8 ± 1.4 ^a^	0.3	<0.001
Chest circumference (cm)	28.6 ± 2.6 ^b^	39.8 ± 2.7 ^a^	1.0	<0.001
Keel length (cm)	11.0 ± 0.6 ^b^	16.8 ± 0.9 ^a^	0.5	<0.001
Drumstick length (cm)	14.5 ± 0.7 ^b^	15.3 ± 0.7 ^a^	0.1	<0.001
Shank length (cm)	10.6 ± 0.4 ^b^	11.4 ± 0.4 ^a^	0.1	<0.001

^a,b^—Means within column and sample with differing superscripts are significantly different (*p* < 0.05).

**Table 2:** Proportion of carcass components in dual-purpose hens and broiler breeder hens after first egg production season.

Trait	Genotype		SEM	*p-*Value
	Dual-Purpose Hens (*n* = 20)	Broiler Breeder Hens (*n* = 20)		
Breast meat (%)	15.3 ± 1.4 ^b^	23.7 ± 1.9 ^a^	0.7	<0.001
Leg meat (%)	18.4 ± 2.4 ^b^	22.5 ± 2.0 ^a^	0.5	<0.001
Skin with subcutaneous fat (%)	14.5 ± 2.0 ^a^	11.2 ± 1.8 ^b^	0.4	<0.001
Abdominal fat (%)	2.1 ± 1.3	2.7 ± 1.3	0.2	0.218
Wings (%)	12.1 ± 0.8 ^a^	9.6 ± 0.5 ^b^	0.3	<0.001
Neck (%)	3.9 ± 0.6	3.6 ± 0.7	0.1	0.102
Carcass remainders (%)	33.7 ± 3.0 ^a^	26.7 ± 3.6 ^b^	0.7	<0.001

^a,b^—Means within column and sample with differing superscripts are significantly different (*p* < 0.05).

**Table 3:** Biometric traits of intestine in dual-purpose hens and broiler breeder hens after first egg production season.

Trait	Genotype		SEM	*p*-Value
	Dual-Purpose Hens (*n* = 20)	Broiler Breeder Hens (*n* = 20)		
Length (cm)				
Total intestine	212.9 ± 0.0 ^b^	254.0 ± 14.5 ^b^	4.1	0.001
Duodenum	32.6 ± 3.7	35.5 ± 3.8	0.6	0.020
Jejunum	67.4 ± 8.2 ^b^	85.2 ± 11.7 ^a^	2.1	<0.001
Ileum	62.3 ± 8.4 ^b^	78.0 ± 8.6 ^a^	2.4	< 0.001
Caeca	40.0 ± 4.2	41.4 ± 6.2	0.8	0.402
Colon	10.6 ± 1.8 ^b^	13.9 ± 1.3 ^a^	0.4	<0.001
Diameter (mm)				
Duodenum	11.3 ± 1.3	12.6 ± 1.3	0.2	0.004
Jejunum	9.9 ± 1.6	11.5 ± 1.3	0.2	0.002
Ileum	8.4 ± 1.5	12.8 ± 1.3	0.2	0.159
Caeca	8.3 ± 1.3	8.7 ± 1.3	0.2	0.355
Colon	9.3 ± 1.3	11.0 ± 2.0	0.3	0.003

^a,b^—Means within column and sample with differing superscripts are significantly different (*p* < 0.05).

**Table 4:** Biometric traits of some internal organs in dual-purpose hens and broiler breeder hens after first egg production season.

Trait	Genotype		SEM	*p*-Value
	Dual-Purpose Hens (*n* = 20)	Broiler Breeder Hens (*n* = 20)		
Proventriculus (g)	8.6 ± 2.1 ^b^	11.1 ± 0.9 ^a^	0.3	<0.001
Gizzard (g)	33.1 ± 5.3 ^b^	38.9 ± 4.7 ^a^	0.9	<0.001
Liver (g)	27.2 ± 6.2 ^b^	69.6 ± 18.2 ^a^	3.9	<0.001
Heart (g)	7.6 ± 2.4 ^b^	16.6 ± 2.1 ^a^	0.8	<0.001
Spleen (g)	2.0 ± 0.6 ^b^	3.8 ± 0.4 ^a^	0.2	<0.001

^a,b^—Means within column and sample with differing superscripts are significantly different (*p* < 0.05).

## Data Availability

Not applicable.
